# Curative Brachytherapy for Inoperable Early-Stage Oesophageal Cancer: A Case Series and Narrative Review

**DOI:** 10.3390/jpm16010013

**Published:** 2025-12-31

**Authors:** Elena Lluzar, Adriana Capdevila, Faegheh Noorian, Antonio Herreros, Cristina Castro, Àngels Gines, Glòria Fernández-Esparrach, Carmen Ares, Yao Qiang, Angeles Rovirosa

**Affiliations:** 1 Faculty of Medicine, Universitat de Barcelona, C/Casanovas 153, 08036 Barcelona, Spain; elluzafr7@alumnes.ub.edu (E.L.); yqiangqi45@alumnes.ub.edu (Y.Q.); 2 Anaesthesia Department, Hospital Clínic-Universitat de Barcelona, C/Villarroel 170, 08036 Barcelona, Spain; acapdevilla@clinic.cat; 3 Radiation Oncology Department, Hospital Clínic Universitat de Barcelona, 08036 Barcelona, Spain; faegheh.noorian@gmail.com (F.N.); herreros@clinic.cat (A.H.); crcastro@clinic.cat (C.C.); mcares@clinic.cat (C.A.); 4 Fonaments Clínics Department, Faculty of Medicine, Universitat de Barcelona, 08036 Barcelona, Spain; 5 Endoscopy Unit, Gastroenterology Department, ICMDM, Hospital Clinic, UB, C/Villarroel 170, 08036 Barcelona, Spain; magines@clinic.cat (À.G.); mgfernan@clinic.cat (G.F.-E.); 6 Departament de Medicina, Facultat de Medicina i Ciències de la Salut, Universitat de Barcelona (UB), C/Casanovas 153, 08036 Barcelona, Spain; 7 Institut d’Investigacions Biomèdiques August Pi i Sunyer (IDIBAPS), C/Casanovas 153, 08036 Barcelona, Spain; 8 Centro de Investigación Biomédica en Red de Enfermedades Hepáticas Digestivas (CIBEREHD), C/Casanovas 153, 08036 Barcelona, Spain

**Keywords:** key early-stage inoperable oesophageal cancer, intraluminal brachytherapy, external beam irradiation, definitive radiotherapy

## Abstract

**Background**: A subset of patients with T1-T2 oesophageal cancer are not candidates for surgery or chemotherapy and have a poor prognosis due to limited treatment options. This study evaluated the combination of external beam radiotherapy (EBRT) and endo-oesophageal brachytherapy (EBT) as a curative treatment in these patients, with cause-specific survival (CSS) and local recurrence-free survival (LRFS) as the primary endpoints. **Methods:** This was a single-centre retrospective analysis of 11 patients with T1-T2 oesophageal cancer treated between 2005 and 2024 with combined EBRT and EBT schedules. Clinical data, treatment schedules, outcomes, and complications were obtained from patient medical records and follow-up documentation. Descriptive statistics and Kaplan–Meier survival analysis were used. **Results:** The median follow-up was 22 months (2–61 months). CSS rates were 79.5% at 2 years, 66% at 3 years, and 30% at 5 years. LRFS rates were 74.1%, 59%, and 39%, respectively. One severe toxicity (grade ≥ 3) was observed. The most frequent mild toxicities were oesophageal mucositis (18.2%) and ulceration (18.2%). **Conclusions**: EBT in combination with EBRT seems to be a feasible and well-tolerated treatment with curative intent for inoperable T1-T2 oesophageal cancer patients, offering favourable survival outcomes in a population with limited therapeutic alternatives.

## 1. Introduction

Oesophageal cancer was ranked as the 11th most common malignancy globally in 2022, with an estimated 510,716 new cases reported. Its lethality remains significant, as it is the seventh leading cause of cancer-related deaths worldwide [1].

The main risk factors for oesophageal cancer in Western countries are gastroesophageal reflux disease, smoking, alcohol consumption, and obesity [2]. This cancer includes two major histological types, squamous cell carcinoma (SCC) and adenocarcinoma, which exhibit distinct epidemiological and clinical characteristics [2]. Clinically, early stages of oesophageal cancer often remain asymptomatic, leading to the detection of most cases in advanced stages. The hallmark symptom of oesophageal cancer is dysphagia, usually accompanied by progressive weight loss [3], although haematemesis, rectal bleeding, melena, and cough can be found in advanced stages [4].

Regarding treatment, endoscopic en bloc resection is the most common treatment option in early disease (cT1, N0, M0), including intraepithelial high-grade dysplasia and most T1 tumours [5]. Contrarily, in T2 and some T1 tumours, the elective treatment is surgery, whereas in more advanced stages, preoperative chemoradiotherapy is the gold-standard treatment [6]. Eligibility for surgery depends not only on the stage of the disease, but also the performance status, age, and comorbidities of the patient and the possibility of preserving quality of life [7]. With the increase in life expectancy, an increase in inoperable cases is expected in the next years due to comorbidities associated with age.

For inoperable patients with T1-T2 tumours, who are unlikely to survive without intervention, radical radiotherapy in combination with high dose rate (HDR) endo-oesophageal brachytherapy (EBT) seems to be a feasible treatment option [8,9]. HDR EBT delivers high doses of radiation directly to the oesophagus wall at the tumour site using specific applicators and a steep dose gradient, minimizing exposure to surrounding healthy tissues [4]. It also shortens the overall treatment time, potentially decreasing tumour cell re-population [10].

One of the main indications of HDR EBT is as a curative treatment for inoperable stage I-II tumours, as it has demonstrated to be particularly effective in relatively small tumours with shallow invasion (length of ≤5 cm, T1 or T2) [11]. Indeed, the American Brachytherapy Society, the Spanish Brachytherapy Group of the Spanish Society of Radiation Oncology (SEOR), the Italian Brachytherapy Society of the Italian Association of Radiotherapy and Clinical Oncology (AIRO), and the Groupe Européen de Curiethérapie and the European Society for Radiotherapy and Oncology (GEC-ESTRO) contemplate curative treatment with EBT alone or combined with external beam radiotherapy (EBRT) as a valid option in non-surgical cases of early oesophageal cancer [4,7,12,13]. However, survival significantly differs depending on tumour depth [14,15], starting from substantial submucosal invasion. According to the GEC-ESTRO Committee, the indications for EBT alone in superficial oesophageal cancer are T1aN0M0, T1bN0M0, and positive margins after mucosectomy without risk factors for oesophageal lymph node involvement, such as G3 and angiolymphatic invasion [12].

The current analysis aimed to assess the outcomes of EBRT combined with EBT as a curative treatment for non-surgical patients with T1, N0, M0–T2, N1, and M0 stage oesophageal cancer. The primary endpoints were cause-specific survival (CSS) and local recurrence-free survival (LRFS) at 2, 3, and 5 years. Secondary endpoints included treatment-related toxicity and tolerability.

## 2. Materials and Methods

This is a retrospective study using a standardized treatment protocol. The study protocol received approval from the Research and Bioethics Institutional Review Board of the Hospital Clinic of Barcelona (HCB/2019/1059). The CEIm approval in 2019 authorized the retrospective analysis and the continued inclusion of subsequent patients treated with the same routine clinical protocol.

### 2.1. Patient Population

The study population included 11 patients with histologically confirmed oesophageal cancer who underwent EBRT plus EBT at our centre between 2005 and 2024.

Tumour staging was performed following the guidelines of the 7th edition of the TNM classification system established by the American Joint Committee on Cancer [16]. Diagnosis confirmation and staging comprised gastroscopy with biopsy and thoraco-abdomino-pelvic computed tomography (CT). Additional staging procedures included endoscopic ultrasound (EUS) in all patients (11/11; 100%), PET-CT in 3/11 (27.3%), EUS-guided fine needle aspiration in 3/11 (27.3%), and bronchoscopy in 1/11 (9.1%), performed to assess potential bronchial infiltration.

Performance status was assessed according to the Karnofsky Performance Status scale and grouped into five categories for analysis, 0 (100%), 1 (80–90%), 2 (60–70%), 3 (40–50%), and 4 (<40%), which corresponded to preserved, slightly reduced, moderately reduced, significantly reduced, and severely impaired functional status, respectively [17]. Most patients were considered inoperable by a multidisciplinary committee due to high surgical risk (10/11; 90.9%) and one refused to undergo surgery (1/11; 9.1%).

Information on tumour characteristics, including size, distance to the dental arcade, and extent of circumferential involvement, were collected from oesophageal endoscopy and EUS reports. Histological type and differentiation grade were retrieved from pathology reports.

### 2.2. Brachytherapy Treatment

Radiation treatment consisted of a combination of EBRT and EBT in all cases, except one patient who only received EBT due to prior radiotherapy for a previous lung cancer. Some patients (3/11; 27.3%) had undergone mucosectomy before treatment with EBT and EBRT.

For treatment planning, patients were referred to the Radiation Oncology Department. Initially, EBRT was performed using a linear accelerator (Linac) after 3D-conformal planning or volumetric modulated arc therapy (VMAT) and EBT was performed with 2D and 3D image-guided planning thereafter. In 2D-planning EBT treatment volumes were defined using information from two coplanar thoracic X-rays and prior oesophagus–gastroduodenal transit studies. The doses administered are shown in Table 1.

The pre-determined interval between EBT fractions was 7 days, with 15 days between EBRT and EBT. Due to an intercurrent disease, in one case, the treatment was administered after 60 days.

In contrast to EBRT planning, CT imaging was acquired with a slice thickness of 1 mm. Images were transferred to the PLATO BPS (Nucletron) before 2014, and Oncentra Brachy (Elekta) after its substitution. The afterloaders used were microSelectron v2 between 2002 and 2018 and microSelectron v3 from 2018 onwards.

Gross tumour volume, clinical target volume, and planning target volume were defined in each CT slice along the length of treatment. Organs at risk (OARs) were also defined in each case (spinal cord, lungs, large vessels, heart and trachea).

**Figure 1:**
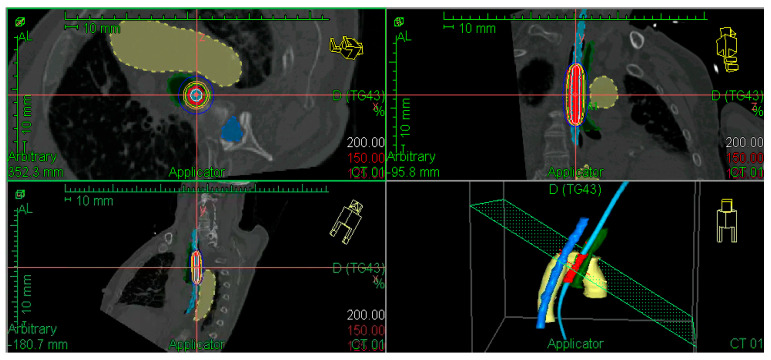
Example of dose distribution in a patient with a previously irradiated T2 tumour in the middle thoracic oesophagus. Aorta in yellow, spinal cord in dark blue, bronchus in green, applicator in light blue, and PTV in red. CTV and GTV are not shown, considering there was a complete response after external beam irradiation.

A dosimetry study was performed to administer the radiation dose at 5 mm from the applicator surface, with an active source length that depended on tumour characteristics and neighbouring OARs. The radiation doses delivered are detailed in the Results Section. The diameter of the applicator was the largest possible in each patient. Figure 1 shows an example of a 3D image-guided EBT dosimetric study.

The EBT procedure was performed as follows: First, oesophageal gastroscopy was carried out under sedation for applicator placement in the Endoscopy Unit at the hospital. A flexible metallic guide was introduced and the gastroscope was subsequently removed while maintaining the position of the guide, allowing the applicator to slide in via a central lumen. The brachytherapy catheter used was the Bonvoisin–Gerard oesophageal applicator.

**Figure 2:**
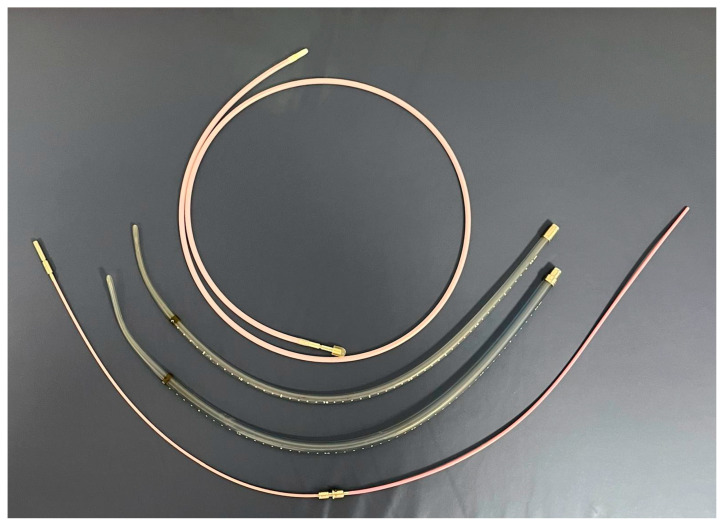
Applicators for endo-oesophageal brachytherapy with diameters of 6 mm, 10 mm, and 13 mm, as well as the transference tube.

The applicator also allowed the introduction of a radioactive source once the dosimetry study had been completed. The usual applicator diameter for EBT was 13 mm. Applicators used in for the patients is shown in Figure 2.

Patients were transferred to a shielded treatment room, where the applicator was connected to the HDR Iridium-192 afterloader. Once correct applicator placement was confirmed, an autoradiography of the treatment plan was performed to verify/confirm the correct positioning of the radioactive source within the applicator, and then the patients were transferred to a shielded room. Thereafter, the treatment was initiated, and when finished, the transference tube and applicator were removed, and the patient then remained in observation for 4–5 h.

### 2.3. Follow-Up

Follow-up was conducted by a multidisciplinary team composed of radiation oncologists, medical oncologists, and general surgeons. It consisted of clinical evaluations, thoracoabdominal CTs, and oesophageal endoscopies (the first being at 3 months). Visits were scheduled 15 days after treatment, monthly for a trimester, and every 6 months thereafter. Ideally, imaging and endoscopy techniques were conducted every 3 months during the first year and annually thereafter, or earlier in cases of suspected relapse or late complications. Side effects such as dysphagia, pain, and weight loss were monitored at each visit.

### 2.4. Endpoints

The primary endpoints of this study were CSS and LRFS, whereas secondary endpoints comprised treatment-related toxicities and overall tolerability.

Response evaluation was based on imaging studies and endoscopic assessments according to the Response Evaluation Criteria in Solid Tumours (RECIST). Complications were assessed using Common Terminology Criteria for Adverse Events (CTCAE) v5 scores. Pain was graded using visual analogue scales, dysphagia was graded according to severity (no, dysphagia to liquids, dysphagia to solids, and aphagia), and body weight evolution was classified as gain, stability, or loss. Follow-up duration was calculated from the date of treatment initiation to the last follow-up or death.

### 2.5. Statistical Analysis

Continuous variables were presented as medians and ranges. Kaplan–Meier curves were constructed to estimate CSS and LRFS. Survival rates were calculated at 2, 3, and 5 years and expressed as percentages of patients at each time interval, and 95% confidence intervals (CI) were calculated for each time point using the Kaplan–Meier method [18]. Statistical analysis was performed using SPSS version 29 (IBM).

## 3. Results

### 3.1. Patient Characteristics

The baseline patient and tumour characteristics are summarized in Table 2. The cohort included 11 patients with inoperable T1 (most being T1b)-2N0-1M0 oesophageal cancer. The median age was 75 years (range: 46 to 90), and the majority were males (7/11; 63.6%). The predominant histological subtype was SCC (9/11; 81.8%). Most tumours were in the middle oesophageal (5/11; 45.5%) or lower oesophageal third (5/11; 45.5%), and clinical staging showed that T1 lesions predominated slightly (6/11; 54.5%) over T2 lesions (5/11; 45.5%). Regarding comorbidities, 5/11 (45.5%) were alcohol consumers and 4/11 (36.4%) were smokers.

Regarding radiotherapy treatment planning, out of the 10 patients who received EBRT, 7 (70%) were treated with a linear accelerator using 3D planning, while 3 (30%) received EBRT treatment using the VMAT technique.

In relation to EBT, 2D planning was performed in three patients (27.3%) and 3D planning in eight patients (72.7%). The applicator diameter was 13 mm in 10 patients (90.9%) and 10 mm in one patient (9.1%). The median active length was 7 cm (range: 5.5 cm to 10 cm).

EBT treatment schedules were individualized and are summarized in Table 2. Two patients did not complete the planned EBT treatment due to toxicity and technical difficulties, respectively.

### 3.2. Follow-Up

The median follow-up time was 22 months (range: 2 to 61 months). According to tumour stage, the median follow-up in T2 vs. T1 patients was 14 months (range: 2 to 46 months), and 30.5 months (range: 4 to 61 months), respectively.

### 3.3. Local Control and Survival

All patients achieved complete response following treatment; however, four patients (36.4%), two T1bN0M0 and two T2N0M0, showed local recurrence during the study period, all detected within 25 months after finishing treatment. One T2N0M0 patient presented distant hepatic metastases 7 months after finishing radiation treatment and died 3 months later.

**Figure 3:**
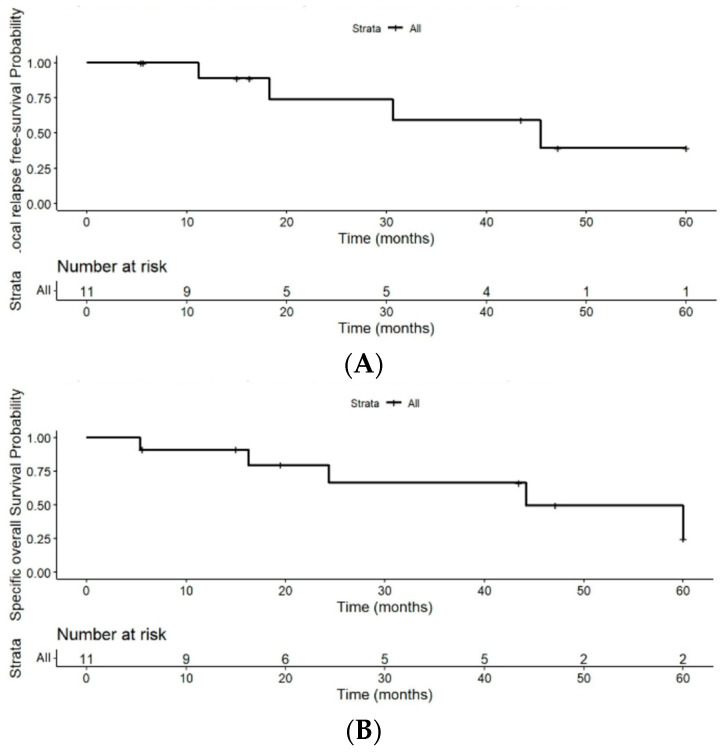
(**A**) Kaplan–Meier curve for cause-specific survival. (**B**) Kaplan–Meier curve for local relapse-free survival despite the limitations of the small number of patients.

The LRFS rates were 74.1% (95% CI: 0.5–1) at 2 years, 59% (95% CI: 0.3–1) at 3 years, and 39% (95% CI: 0.1–1) at 5 years (Figure 3B). Among patients who developed local relapse (4/11; 36.4%), the median time from diagnosis to relapse was 32.5 months (range: 19 to 45 months). These results are summarized in Table 3.

CSS rates in the 11 patients were 79.5% (95% CI: 0.6–1) at 2 years, 66% (95% CI: (0.4–1) at 3 years, and 30% (95% CI: 0.05–1) at 5 years of follow-up (Figure 3A).

Out of the 11 patients, 5 (45.5%) died due to the disease, 4 (36.4%) died from non-cancer-related disease, and 2 (18.2%) patients were alive and disease-free at the time of analysis. Among patients who died from the disease (5/11; 45.5%), the median time from diagnosis to death was 24 months (range: 5 to 50 months).

### 3.4. Treatment Toxicity

HDR EBT was generally well tolerated. Acute complications were not seen in 8/11 (72.7%) patients. There were 2/11 (18.2%) cases of grade 1 and 2 oesophageal mucositis, both of which appeared within 10 days after treatment initiation and were observed to heal within one month. One patient presented with a grade 5 tracheoesophageal fistula and died shortly after by pneumonia and mediastinitis. This patient had received 45 Gy of EBRT and 6 Gy of EBT; however, a posterior revision of his CT revealed the previous existence of the fistula before EBT.

This was, therefore, an isolated case and not directly caused by radiation treatment; thus, it was considered the worst grade observed and clearly stated to be attribution grade 5 (unrelated) according to CTCAE v5.

Of the 11 patients, 4 (36.4%) developed late toxicities after treatment, all of which were ≤grade 2. Ulceration of grade 1 and 2 occurred in two patients (18.2%). In addition, a single case of grade 1 radiation-induced oesophagitis (1/11; 9.1%) and one grade 2 candidiasis-related oesophagitis (1/11; 9.1%) were observed. The latter and one of the ulcers resolved over time, while the other two complications were still present at the last follow-up.

Dysphagia after radiation treatment was only observed in the patients who presented with toxicities or local relapses. One patient presented weight loss related to anorexia (reduced appetite), whereas the rest remained stable or improved in two cases. No patient referred pain related to treatment.

## 4. Discussion

In the current study, EBT in combination with EBRT showed promising local control and survival rates for early-stage oesophageal carcinoma. The 2-, 3-, and 5-year CSS rates were 79.5%, 66%, and 30%, and the LRFS rates were 74.1%, 59%, and 39%, respectively. Complete response was achieved in 100% of patients.

The European Society of Gastrointestinal Endoscopy recommends brachytherapy as a valid alternative (strong recommendation, high-quality evidence) alone or in addition to stenting in oesophageal cancer patients with malignant dysphagia and a longer expected life expectancy [19]. Doses of 50–64 Gy offer local control of 45–55%, and the OAR limit can cause a decrease in the dose. Brachytherapy allows the dose to be increased mainly in the endoluminal component of the tumour [20,21]. The AIRO society performed a systematic review which showed that EBRT + BT was the best treatment for inoperable T1 oesophageal cancer [7]. Despite different recommendations and guidelines, EBT is a treatment that is not extensively used in the medical community, mainly due to a lack of applicators and experience in the brachytherapy units and preferences of each hospital regarding the available techniques [10]. A summary of the previous studies on the topic and their main results is shown in Table 4.

Considerable diversity among the series can be observed, with 5-year overall survival (OS) rates ranging from 35% to 84% and CSS rates from 38 to 97%. These discrepancies likely reflect heterogeneity in study populations and methodologies, such as tumour stage (T1a vs. T1b vs. T2), cohort sizes, differences in radiation schedules, and the possible addition of chemotherapy. Therefore, direct comparison should be interpreted with caution due to differences in outcome definitions and treatment protocols.

Regarding treatment dosing, the Spanish Brachytherapy Group of the SEOR recommends a regime of two fractions of 5 Gy at 5 mm of the applicator surface after 45–50 Gy of EBRT [4], a regime generally followed in our case series, with slight variations.

A recent study conducted by Ye et al. in 2022 [27] compared the efficacy and side effects of EBRT alone or in combination with EBT as a curative treatment for oesophageal cancer, in a cohort of 64 T1-T3N noM0 patients who received either treatment. The EBRT dose was 50 Gy, and the EBT dose was 10 Gy (5 Gy × 2 fractions prescribed at 5 mm of the applicator surface). At three years, the LRFS rates of EBRT + EBT vs. EBRT alone were 25% and 9%, and the OS rates were 38% and 9%, respectively. Additionally, a study conducted by Ishikawa et al. [20] in 2010 showed a statistically significant difference in the 5-year CSS rate for T1 patients receiving EBRT + EBT vs. EBRT alone (86% vs. 62%, *p* = 0.04).

These outcomes differ somewhat from those observed in our cohort, possibly due to the inclusion of more advanced (Ye et al.) or earlier (Ishikawa et al.) tumour stages. These studies support the added value of EBT in improving local control and survival in this setting of patients without any other possible curative treatment. Our findings are consistent with this trend, although further studies with larger cohorts are needed to confirm these results.

Interestingly, some series restricted to T1 tumours obtained much higher survival outcomes. In 2012, Tamaki et al. studied 54 T1 patients who received 56–60 Gy doses of EBRT and 9–10 Gy of EBT in two fractions prescribed at 5 mm of the applicator, obtaining a five-year CSS of 85%, locoregional control of 75% and complete response of 80% [26]. Similarly, Murakami et al. [14] performed a univariate analysis that showed that tumour depth was a significant predictor for CSS (*p* < 0.001), locoregional control (*p* = 0.02), and OS (*p* < 0.001). The study included 87 T1 patients irradiated with 45–46 Gy of EBRT and 10–15 Gy of EBT (2 fractions prescribed at 5 mm of the applicator surface), showing a 5-year CSS and locoregional control of 97% and 75% for T1a tumours, and 55% and 49% for T1b tumours. Although the outcomes are slightly better than those obtained in our study, this difference may be partially explained by the presence of a 45.5% T2N0M0 patient population in our cohort. Nonetheless, our follow-up time according to tumour depth is in line with their analysis, since the median follow-up time in our cohort was 14 months (range: 2 to 46 months) for T2 tumours, 30.5 months (range: 15 to 42 months) for T1b, and 61 months (one case) for T1a tumours.

Regarding toxicity, the treatment was generally well tolerated and in agreement with other series as shown in Table 4. All complications were of grade ≤ 2, the most frequent being oesophageal mucositis (18.2%) and ulceration (18.2%). Additionally, there were two individual cases of radiation-induced oesophagitis (9.1%) and candidiasis-related oesophagitis (9.1%). No treatment-related complications of grade 3 or higher were observed. Considering that one case died due to an oesophageal fistula, these patients should be very well examined before EBT.

The favourable outcomes and low toxicity observed in our study align with the results reported by Okawa et al., (1999) [8]. Their analysis found a statistically significant difference in the 5-year CSS rate when comparing patients with a tumour length ≤ 5 cm who received EBRT + EBT to the whole EBRT + EBT cohort (64% vs. 38%; *p* = 0.025). All the tumours in our study measured ≤ 4 cm, consistent with prior evidence suggesting that smaller lesions respond particularly well to combined radiotherapy. Acute and late ulcerations can be associated with large linear ulcerations and can develop fistulas, but this is very unusual when the dose limits are respected. Other factors are associated with toxicity are the EBT dose administered. In this setting, doses superior to 18 Gy have been related to an increase in stenosis and fistulas in the past. Haemorrhage is not a common complication in T1-T2 tumours. In the present series, EBRT and EBT doses did not induce relevant complications and provided a relatively good life expectancy [28]

Our cohort included one patient treated with exclusive EBT due to previous irradiation of lung cancer. He received a three-fraction of 7.5 Gy each scheme prescribed at 5 mm of the applicator surface, experienced no early or late toxicity, and is alive and disease-free at the time of analysis. These findings are consistent with previous reports showing encouraging outcomes with EBT alone in early-stage oesophageal cancer [29,30,31].

Although surgery is the standard curative treatment for oesophageal cancer, a subset of elderly patients with concomitant comorbidities are not eligible candidates for both surgery and chemotherapy. Individuals deemed inoperable due to comorbidities tend to have a notably worse prognosis and die due to limited treatment options. In this scenario, it is crucial to consider alternative treatments. Brachytherapy, particularly when combined with EBRT, has shown to be an effective technique, especially in T1-T2 tumours of ≤5cm in length, which may represent a critical therapeutic window with an acceptable low rate of complications and favourable survival outcomes, achieving rates of up to 97% of CSS at 5-years in T1a tumours. Along this line, randomized trials between definitive combined radiotherapy and surgery in T1-T2 oesophageal tumours are required. Choe et al. performed a meta-analysis of T1 oesophageal cancers in 525 patients. Of these, 325 were treated with EBRT + EBT and the rest only received EBRT. Interestingly, in the EBRT-only subgroup, locoregional control rates were 9% higher, at 91% (95% CI, 76–100%), at 1 year, but lower at 3 years, at 76% (95% CI 67–85%), and at 5 years, at 72% (95% CI 57–85%), in comparison to EBRT + EBT therapy. The authors concluded that the level of evidence was IC (strong recommendation, low-quality evidence) for EBRT + EBT [32].

Finally, given the favourable outcomes obtained in the patients who received exclusive EBT, together with previous supporting evidence, further investigation into the role of EBT alone as a curative treatment in early oesophageal carcinoma is encouraged. In a study of inoperable stage I oesophageal cancer performed in 2006, Ishikawa et al. compared an EBRT treatment group with an EBRT boosted with brachytherapy group. The locoregional control rate was 69.5% with EBT alone as compared to 79.5% with combined EBRT + EBT therapy, demonstrating a 10% increase. The rates were statistically significant for this study, but interestingly, there was no difference in survival benefits [33].

In a meta-analysis by the AIRO Society on 514 T1 cancer patients, the median local control was 77% (63–100%), disease-free survival (DFS) was 68.4% (range 49–86.3%), OS was 60% (range 31–84%), CSS was 80% (range 55–100%), and grade 3–4 toxicity ranged from 0 to 26% [7]. Considering the above-mentioned results, those of the present series are in the line with what is to be expected, but lower results should be obtained in T2 cases.

### Limitations

This study had some limitations such as the small sample size that limits the statistical power of the analysis and extrapolation of the findings, as well as the retrospective nature of the study which may also influence the outcomes observed. Nevertheless, when considered by stages, the results are similar to those reported by other series. The relatively high proportion of T2 (45.45%) tumours and the improvements in planning techniques over time may have impacted survival and toxicity outcomes. Therefore, future prospective studies with larger and more homogeneous cohorts are needed to validate these findings.

## 5. Conclusions

Combined schemes of EBRT and HDR EBT represent effective alternatives for patients with inoperable early-stage oesophageal cancer. This treatment is generally well tolerated and offers favourable survival outcomes in patients with limited therapeutic options. Given the increasing number of inoperable patients, mainly due to advanced age and comorbidities, it is essential to establish the benefits of combined EBRT and EBT through prospective randomized trials.

## Figures and Tables

**Table 1:** Radiotherapy schemes and aspects of treatment planning.

PATIENT	PREVIOUS MUCOSECTOMY	SEQUENCE	EBRT DOSE (GY)	EBRT FRACTIONS	EBT (GY)	EBT FRACTIONS	EBT DOSING INTERVAL (DAYS)	EBT PLANIFICATION (2D, 3D)	ACTIVE SOURCE LENGTH (CM)	APPLICATOR DIAMETER (MM)	(LARGER TUMOUR SIZE (CM)	EQD2 (A/B = 10) TUMOUR	EQD2 (A/B = 3) MUCOSA
**1**	No	EBRT → EBT	45	25	6	1	-	2D	10	13	5	52.3	74.7
**2**	No	EBT → EBRT	61.2	34	10	2	7	2D	6	13	2	72.7	107.8
**3**	Yes	EBRT → EBT	59.4	33	12	2	60	2D	Unknown	13	4	74.4	Unknown
**4**	Yes	EBRT → EBT	50.4	25 + boost	10	2	7	3D	7	13	3	62.1	96.1
**5**	Yes	EBT →EBRT	45	25	10	2	7	3D	7	13	3	56.8	91.0
**6**	Yes	EBT → EBRT	52.6	25 + boost	5	1	-	3D	7	13	3	58.3	75.2
**7**	No	EBRT → EBT	45	25	10	2	7	3D	Unknown	13	1	56.8	Unknown
**8**	No	EBRT → EBT	50.4	28	10	2	7	3D	7	13	3	62.1	96.1
**9**	No	EBRT → EBT	50	28	15	3	7	3D	9	13	4	67.9	117.0
**10**	Yes	EBRT → EBT	54	30	10	2	7	3D	6	10	2	65.6	112.4
**11**	No	EBT	No		22.5	3	3–7	3D	5.5	13	1.5	32.8	74.6

EBRT = external beam radiotherapy; EBT = endo-oesophageal brachytherapy.

**Table 2:** Patient and tumour characteristics.

	*Patients*	
*Characteristic*	Number	**%**
**Age (years)**		
46–65	3	27.3
66–75	3	27.3
76–85	4	36.4
>85	1	9.1
Median	75	
Range	46–90	
*Sex*		
Male	7	63.6
***Female***	4	36.4
**Performance status (KPS)**		
0	1	9.1
1	8	72.7
2	1	9.1
3–4	0	0.0
Unknown	1	9.1
*Comorbidities*		
Smoker	4	36.4
Enol	5	45.5
Diabetic	3	27.3
Hypertension	5	45.5
Dyslipidemia	3	27.3
Obesity	1	9.1
Neoplasia ORL	0	0.0
Barret	3	27.3
Other	8	72.7
*Stage*		
T1	6	54.5
T2	5	45.5
N0	10	90.9
N1	1	9.1
*Localization*		
Upper third	1	9.1
Middle third	5	45.5
Lower third	5	45.5
Size (cm)		
<2	3	27.3
2	4	36.4
3	3	27.3
4	1	9.1
*Histology*		
Adenocarcinoma	2	18.2
quamous cell	9	81.8
Grade		
Well-differentiated	4	36.4
Moderately differentiated	5	45.5
Poorly differentiated	1	9.1
Unknown	1	9.1

KPS = Karnofsky Performance Status; Enol: alcohol consumption.

**Table 3:** Disease-specific survival and local relapse-free survival at 2, 3, and 5 years were estimated using the Kaplan–Meier method with 95% confidence intervals.

*Survival Measure*	*2-Year (%) [95% CI]*	*3-Year (%) [95% CI]*	*5-Year (%) [95% CI]*
*Disease-specific survival*	79.5% [0.6–1]	66% [0.4–1]	30% [0.05–1]
*Local relapse-free survival*	74.1% [0.5–1]	59% [0.3–1]	39% [0.1–1]

CI = confidence interval.

**Table 4:** Summary of previous evidence on the combination of EBT and EBRT as an intention-to-cure treatment in early oesophageal carcinoma.

*Study*	*N*	*T (TNM)*	*EBRT Dose (Mean, Gy)*	*EBT Mean* *Dose (Gy)*	*Chemotherapy*	*Complete* *Response* *(%)*	*Outcomes (Main Endpoints)*	*Complications*
*Yorozu et al. (1999)* [22]	124	T1-T2	40–61	8–24	Yes (41%)	73	LCR: 74% stage I, 35% stage II (EBRT + EBT) OS (2y): 71% stage I, 31% stage II (EBRT + EBT)	Late ulceration and stenosis: 12% Late, severe: 2.5%
*Murakami et al. (1999)* [23]	32	T1-T2	50–66	10–12	Yes (32%)	100	LRC (3y): 70% T1, 83% T2 OS (3y): 83% T1, 51% T2	Ulcer (8/32; 20%), stenosis (2/32; 6.3%).
*Okawa et al. (1999)* [8]	43	T1-4N0-1	60	10	Yes	56	CSS (5y): 38%	-
*Pasquier et al. (2006)* [24]	63	T1	57.1	8.82	Yes	98	CSS (5y): 76.9% DFS (5y): 54.6% OS (3y, 5y, 7y): 57.9%, 35.6%, 26.6%.	Late, severe: oesophageal stenosis (6/66; 9.1%)
*Yamada et al. (2006)* [25]	63	T1	55–60	10–12	Yes		CSS(5y): 76.3% DFS (5y): 63.7% OS (5y): 66.4%	Late grade ≥ 4: oesophageal fistula (2/63; 3.2%). Ulcers or stenosis (10%)
*Ishikawa et al. (2010)* [20]	36	T1	60	9–10	No	87	CSS (5y): 86% LCR (5y): 75%	Ulcers: 5/36 (13.9%) Grade ≥ 2: cardiorespiratory (2/36: 5.6%)
*Tamaki et al. (2012)* [26]	54	T1	56–60	9–10	No	80	CSS (5y): 86% OS (5y): 61% LRC (5y): 79%	Grade ≥ 2: 5/54 (9.3%)
*Murakami et al. (2012)* [14]	87	T1	45–46	10–15	No	83	CSS (5y): 97% mucosal and 55% submucosal OS (5y): 84% mucosal and 31% submucosal LRC (5y): 75% mucosal and 49% submucosal	Acute grade ≥ 3 acute: oesophagitis (2/87; 2.3%), leucopenia (1/87; 1.1%) Late grade ≥ 3: ulcer (5/87; 5.7%), cardiac ischemia and heart failure (9/87; 10.3%), pneumonitis (4/87; 4.6%), cardiac (10/87; 11.5%), fistulas (2/87; 2.3%), ulcers (3/87; 3.4%)
*Ye et al. (2022)* [27]	64	T1-3N M0	50	10	No	66	LRFS (3y): EBT + EBRT 31% OS (3y): EBT + EBRT 38% LRC (3y): EBT + EBRT 25%	Acute grade ≥ 3: dysphagia (2/64; 3.1%). Late: fistula (3/64; 4.7%), radiation pneumonia (3/64; 4.7%), stenosis (5/64; 7.8%)
*Present series* *(2025)*	11	T1-T2	51.5	10	No	100	CSS (3y, 5y): 66%, 30%. LRFS (3y, 5y): 59%, 39% Median follow-up: T1a: 61months T1b: 30.5 months (15–42) T2: 14 months (2–46)	Acute: oesophageal mucositis (2/11; 18.2%) Late: ulceration (2/11; 18.2%)

EBRT = external beam radiation therapy; EBT = endo-oesophageal brachytherapy; LCR = locoregional control; OS = overall survival; CSS = cause-specific survival; DFS = disease-free survival; LRFS = local relapse-free survival.

## Data Availability

The original contributions presented in this study are included in the article. Further inquiries can be directed to the corresponding author.
